# Enhancing human activity recognition for the elderly and individuals with disabilities through optimized Internet-of-Things and artificial intelligence integration with advanced neural networks

**DOI:** 10.3389/fninf.2024.1454583

**Published:** 2024-11-19

**Authors:** R. Deeptha, K. Ramkumar, Sri Venkateswaran, Mohammad Mehedi Hassan, Md. Rafiul Hassan, Farzan M. Noori, Md. Zia Uddin

**Affiliations:** ^1^Department of Information Technology, SRM Institute of Science and Technology, Chennai, Tamilnadu, India; ^2^Department of Computer Science and Engineering, Rajalakshmi Institute of Technology, Chennai, Tamilnadu, India; ^3^Department of Artificial Intelligence and Data Science, Rajalakshmi Institute of Technology, Chennai, Tamilnadu, India; ^4^Department of Information Systems, College of Computer and Information Sciences, King Saud University, Riyadh, Saudi Arabia; ^5^Department of Computer Science, Central Connecticut State University, New Britain, CT, United States; ^6^Department of Informatics, University of Oslo, Oslo, Norway; ^7^Department of Sustainable Communication Technologies, SINTEF, Oslo, Norway

**Keywords:** human activity recognition, Internet of Things, artificial intelligence, gated recurrent networks, deep extreme feedforward neural networks, artificial water drop optimization

## Abstract

Elderly and individuals with disabilities can greatly benefit from human activity recognition (HAR) systems, which have recently advanced significantly due to the integration of the Internet of Things (IoT) and artificial intelligence (AI). The blending of IoT and AI methodologies into HAR systems has the potential to enable these populations to lead more autonomous and comfortable lives. HAR systems are equipped with various sensors, including motion capture sensors, microcontrollers, and transceivers, which supply data to assorted AI and machine learning (ML) algorithms for subsequent analyses. Despite the substantial advantages of this integration, current frameworks encounter significant challenges related to computational overhead, which arises from the complexity of AI and ML algorithms. This article introduces a novel ensemble of gated recurrent networks (GRN) and deep extreme feedforward neural networks (DEFNN), with hyperparameters optimized through the artificial water drop optimization (AWDO) algorithm. This framework leverages GRN for effective feature extraction, subsequently utilized by DEFNN for accurately classifying HAR data. Additionally, AWDO is employed within DEFNN to adjust hyperparameters, thereby mitigating computational overhead and enhancing detection efficiency. Extensive experiments were conducted to verify the proposed methodology using real-time datasets gathered from IoT testbeds, which employ NodeMCU units interfaced with Wi-Fi transceivers. The framework's efficiency was assessed using several metrics: accuracy at 99.5%, precision at 98%, recall at 97%, specificity at 98%, and F1-score of 98.2%. These results then were benchmarked against other contemporary deep learning (DL)-based HAR systems. The experimental outcomes indicate that our model achieves near-perfect accuracy, surpassing alternative learning-based HAR systems. Moreover, our model demonstrates reduced computational demands compared to preceding algorithms, suggesting that the proposed framework may offer superior efficacy and compatibility for deployment in HAR systems designed for elderly or individuals with disabilities.

## 1 Introduction

In a recent survey, the World Health Organization (WHO) highlighted that approximately 650 million individuals of active age globally live with disabilities (Chen et al., 2020). There exists an urgent need for adequate facilities to accommodate these individuals effectively. The deployment of human activity recognition (HAR) systems has seen a rapid increase across various fields, including healthcare, Smart home technology, and crime behavior identification (Chen et al., 2020). These systems are implemented with the dual objectives of enhancing the quality of life and fostering individuals' independence (Yao et al., 2021; Kushwaha et al., 2021). Additionally, HAR technologies are increasingly utilized to support the elderly and individuals with disabilities in monitoring their physical performance post-treatment, aiming to elevate their living standards significantly.

HAR serves as a critical intermediary between human-centric activities and detection mechanisms. Presently, the incorporation of artificial intelligence (AI) algorithms with the Internet of Things (IoT) is employed to forge effective HAR systems to assist the elderly or individuals with disabilities (Lester et al., 2005; Nasir et al., 2021). The marked preference for deep learning (DL) over ML has propelled HAR systems into a realm of enhanced performance. Notably, convolutional neural networks (CNNs) (Nweke et al., 2018; Ramasamy Ramamurthy and Roy, 2018), recurrent neural networks (RNN) (Ranasinghe et al., 2013, 2016), and long short-term memory (LSTM) networks are instrumental in the advancement of HAR systems (Alharbi et al., 2021).

Furthermore, research interest in hybrid DL approaches is growing, focusing on the development of sophisticated HAR systems for individuals with disabilities (Saleh and Hamoud, 2021; Moon et al., 2020; Munoz-Organero and Ruiz-Blazquez, 2017). Despite the high accuracy of these models in identification tasks, their intricate preprocessing and framework design can introduce significant complexity and latency, particularly critical in emergency scenarios (Jiang and Yin, 2015; Laput and Harrison, 2019). Thus, the design methodology for HAR systems must maintain a judicious balance between performance and complexity, ensuring the deployment of systems with manageable computational demands (Ha and Choi, 2016).

In response to these issues, this article introduces a pioneering ensemble of GRN and deep extreme feedforward neural networks (DEFNN) amalgamated with artificial water drop optimization (AWDO) to optimize assistance performance while curbing complexity. This study aims to develop intelligent HAR systems for individuals with disabilities, facilitating autonomous data collection and activity classification by harmonizing IoT and DL technologies (Shen et al., 2018; Mekruksavanich and Jitpattanakul, 2021). The principal offerings of this study are delineated as follows:

1. The deployment of gated recurrent neural networks (RNNs) coupled with dense feedforward networks to realize HAR systems that are both computationally efficient and highly effective.
2. Adopting the artificial water drop algorithm to fine-tune the hyperparameters within the training network, thus optimizing learning durations and diminishing computational burdens. This nature-inspired algorithm is posited as a novel alternative to conventional optimizers in learning frameworks.
3. The establishment of real-time IoT test beds, as delineated within this article, for robust data acquisition that encapsulates a spectrum of human activities.
4. The formulation of diverse evaluation metrics, the results of which are benchmarked against extant DL-based HAR systems.

The subsequent structure of the document is as follows: Section 2 elucidates interrelated articles from various scholars. Section 3 explicates the proposed approach. Section 4 is dedicated to showcasing the experimental outcomes and comparative analysis. Finally, Section 5 furnishes conclusions and outlines prospects for the future research.

## 2 Related works

Recent research contributions to HAR unveil significant advances in employing machine learning and deep learning paradigms to enhance the standard of living for individuals, especially those with disabilities. This includes the development of a deep transfer learning-based HAR device designed to aid individuals with disabilities, emphasizing the role of data preprocessing in improving recognition accuracy (Fotouhi et al., 2022; Zhang et al., 2021). Despite its merits in enhancing accuracy, the model is critiqued for its lengthy training periods, which may limit its real-time application (Mihoub, 2021; Sangeetha et al., 2023).

This study presents a sensor-driven HAR method that employs a deep learning framework incorporating a novel inverted attention mechanism grounded in transformer architecture, aimed at fine-tuning the learning process (Achirei et al., 2022). While this method is notable for its improved learning rates and enhanced attention module calibration, it is also equally plagued by prolonged training durations, echoing the concerns raised by Karayaneva et al. (2023).

LSTM networks are used to assess information sourced from IoT devices in smart homes, enabling real-time HAR. The approach is notable for its ability to predict subsequent activities accurately, yet it is marred by significant computational complexities, which could hinder its scalability and broader application (Alotaibi et al., 2023).

A three-dimensional (3D) ResNet with a multistage fusion technique is proposed that demonstrate a sophisticated approach to HAR that achieves high training efficiency and accuracy. However, its practical application is limited due to its dependence on specific data types and the challenges it faces in real-time environments (Pramanik et al., 2023).

An RNN-based HAR system for smart homes employs various strategies to comprehensively analyze the feature space. Despite identifying an effective categorization method, the system's effectiveness is constrained by its limited capability to process large datasets, indicating a gap in its adaptability (Uddin and Soylu, 2021) in recognizing human activities through feature analysis and a deep neural network (DNN) classifier. Their focus on body behaviors and comprehensive data recognition is promising. However, the slow processing speed is a significant limitation, potentially affecting the user experience and the timeliness of the HAR output (Duhayyim, 2023).

While these above studies push the boundaries in terms of technology use and application areas, they all have common drawbacks, such as high computational costs, raising questions about their feasibility in resource-constrained scenarios (Roy and Cheung, 2018).

Finally, Roy and Cheung (2018) explored a multimodal HAR system that employs LSTM and neural structured learning (NSL) with wearable sensors, notable for its robust modeling of time-sequential data. By utilizing non-linear generalized discriminant analysis for feature extraction, HAR system can simulate various human activities, offering improvements in accuracy and processing speed; however, further investigation is required on the scalability of this system and its effectiveness with large datasets (Priyanga et al., 2021).

### 2.1 Limitations

- Recognition accuracy and lack of complexity are the prime limitations of IoT-based HAR technology; however, it is gaining significant attention due to its low cost.
- DNN exhibits slow processing speed, which stands as a significant limitation, potentially affecting the HAR output's user experience and timeliness.
- One-dimensional convolutional neural network model (1D-CNN) and DNN face challenges in retaining relevant historical information, leading to the well-known vanishing gradient problem.
- The LSTM network models undermine the network's ability to learn from long sequences, affecting the reliability of the results in real-time systems.

### 2.2 Research gap

- The prime drawback of these technologies remain to be integrating sensors in the home environment for continuous monitoring (see Table 1). For example, an apartment can have many residents, where monitoring their individual activities become more challenging.
- Vision-based activity recognition: It is challenging to detect activity when live recordings are streamed through cameras for vision-based activity recognition.
- Different classification algorithms are precise, and time-consuming, and yield better results. It is known that low computational complexity algorithms perform worse in terms of accuracy as compared to algorithms with high computational complexity.

**Table 1:** Limitations of previous work and performance metrics.

**References**	**Proposed method**	**Limitation**	**Performance metrics**
Huang et al. (2006)	Extreme learning machine (ELM) in single hidden layer feedforward neural networks (SLFNs)	Time complexity	Precision, 89.5%; Recall, 89.5%; Accuracy, 89.5%
Wang et al. (2015)	Parallel online sequential	Analysis of the large data	Precision, 88%; Recall, 87%; Accuracy, 90%
Xin et al. (2021)	Artificial raindrop algorithm	Inapplicable process	Accuracy, 95%
Choudhury and Soni (2023)	CNN–LSTM	Raw-sensor data is challenging	Detection rate, 93.5%
Jiao and Zhang (2023)	CNN	HAR is low quality and cost	Accuracy, 99.4% and 99.0%
Arzani et al. (2021)	Probabilistic graphical models (PGMs)	Recognition is the diversity	Accuracy, 92.4%
Dahal and Moulik (2024)	Multilayer stacking	Complexity predicts	Accuracy, 96.4%
Yin et al. (2024)	LSTM	General rationales	Accuracy, 98.4%
Sharma et al. (2024)	CNN	Contrastive loss	Accuracy, 99.5%
Zhu and Sheng (2012)	Dynamic Bayesian Network (DBN)	Complex daily activities	Detection rate, 99.8%
Helmi et al. (2021)	Gradient-based optimizer (GBO)–gray wolf optimizer (GWO)–support vector machine (SVM)	Potentially high computational cost	Accuracy-98%, precision- 98.12%
Wang et al. (2024)	Multilayer perceptron (MLP)-like architecture	Generalization to diverse activity types	Accuracy, 98.61% on wireless sensor data mining (WISDM) data and 90.41% on OPPORTUNITY data
Zhang et al. (2023)	Attention-based bidirectional long short-term memory (BiLSTM)	Higher algorithmic complexity	Accuracy, 98.37%; F1-score, 98.42%
Jaén-Vargas et al. (2022)	CNN–LSTM	Fixed frequency, only three activities	Accuracy, 998.8%; F1-score, 82.80%

## 3 Proposed model

**Figure 1:**
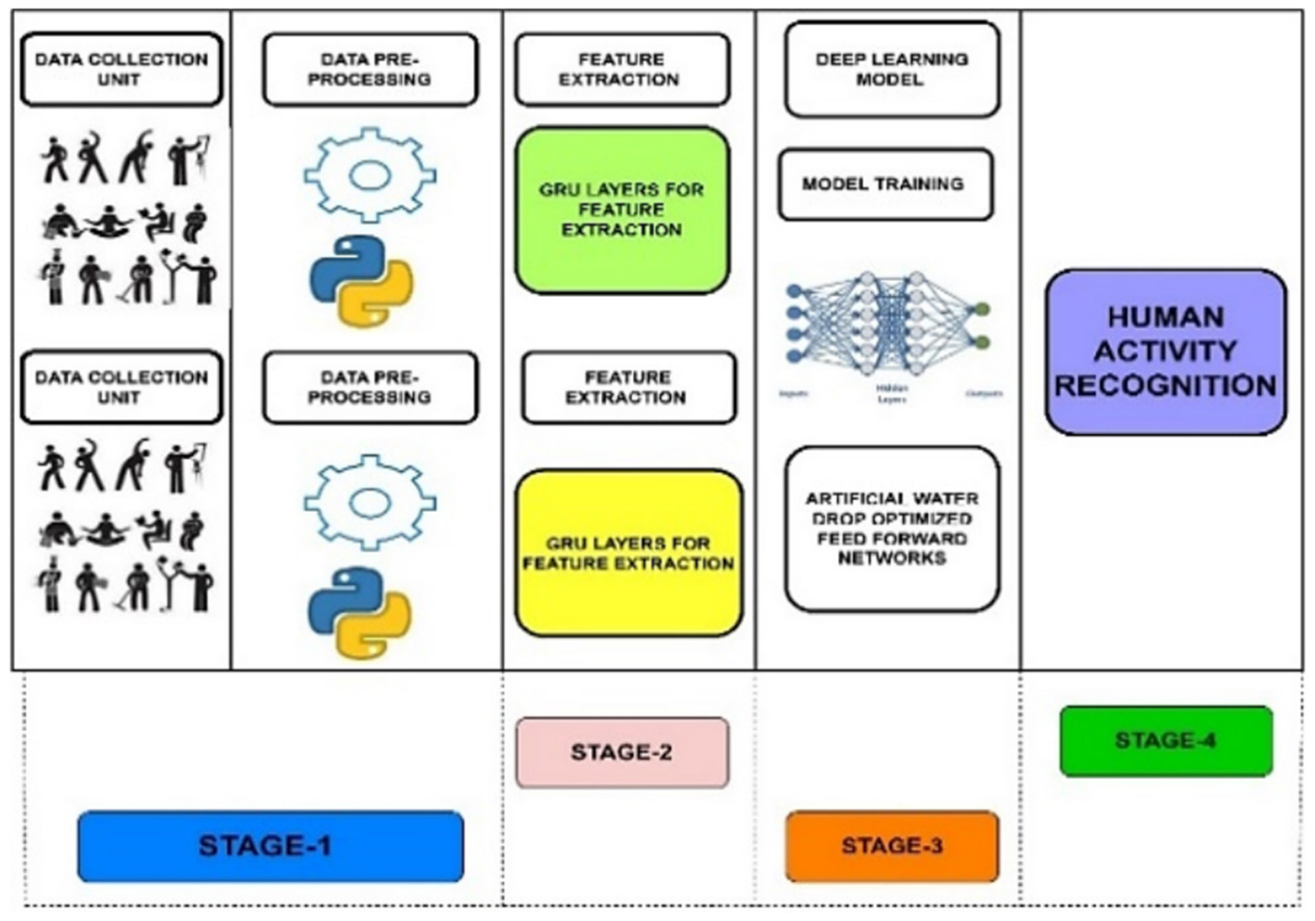
Proposed framework for intelligent HAR system using IoT and deep learning algorithm.

In this study, we introduce a novel hybrid deep learning-based HAR system intended to identify activities for individuals with disabilities, aiming to enhance their quality of life. The suggested methodology, as depicted in Figure 1, comprises four primary stages: (i) IoT unit, (ii) preparation of information, (iii) attribute extraction using proposed GRN units, and (iv) activity recognition.

### 3.1 IoT-based data collection process

**Figure 2:**
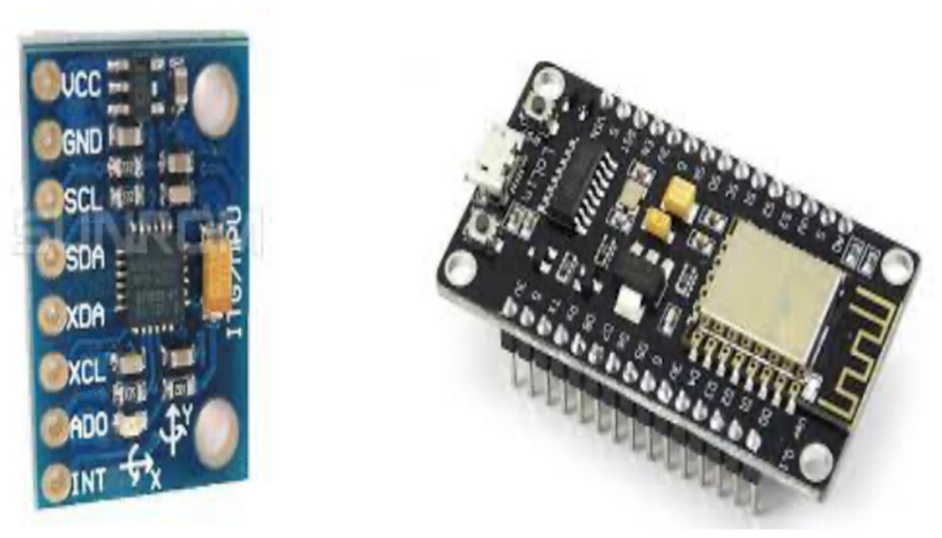
Sensors and microcontrollers used for experimentation.

We enlisted ~50 volunteers with body weights varying from 30 to 65 kg. IoT gadgets powered by batteries collected data of their body movements. Table 2 delineates the sensors, microcontrollers, and cloud technologies utilized for the data collection. The data collection was facilitated by ADXL435 three-axis accelerometers (Figure 2) and BMG250 three-axis gyroscopes connected to NodeMCU *via* MCP3008 (10-bit analog to digital converters [ADC]). MicroPython programming enabled data transmission to the cloud. Lithium–ion battery (Li-ion) battery series, which are replaceable upon power depletion, powered the boards.

**Table 2:** The hardware specifications required for an efficient data collection unit.

**S. No**.	**Hardware used**	**Specifications**	**Application**
01	Node MCU	Main system on chip unit (SOC)	Used for processing the input data
02	MCP3008	10-bit ADC with 8 input channels	Converting the analog sensors to digital values
03	ADXL435	Three-axis accelerometers	Calculates the subject's activity with consideration of human activity
04	BMG250	Three-axis gyroscopes are used	

**Figure 3:**
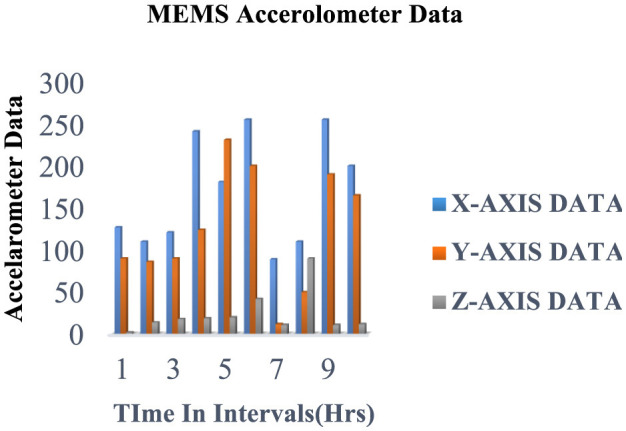
Data samples obtained from hand movements (accelerometers) utilizing IoT experimentation environments.

**Figure 4:**
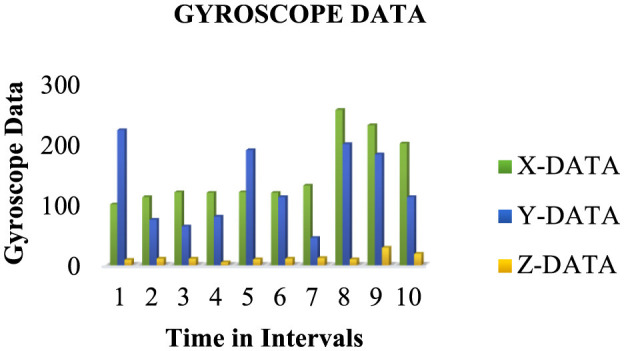
Data samples gathered for manual movements (accelerometers) utilizing IoT experimentation platforms.

For about 2.5 months, we amassed a notable 945,903 samples capturing various activities such as eating, walking, and lying, which were vital in assessing ' physical activity levels and detecting falls of individuals with disabilities. Each subject performed four distinct activities for 2 min at a 15-Hz sampling rate, generating 50 data points per second. Sensor data were stored in the cloud, structured within data frames, as outlined in Table 3, and then downloaded for subsequent processing. Figures 3, 4 depict the data distribution over time.

**Table 3:** Detailing of the properties associated with the information secured in the cloud.

**S. No**.	**Attributes obtained**	**Description**
01	Subject ID	Depicts the information of the subjects
02	Sensors X1, Y1, and Z1	Sensors obtained from the accelerometers
03	Sensors X2, Y2, and Z2	Sensors obtained from Gyroscopes
04	Activity label, L	L has been labeled from 0 to 4

### 3.2 Data preprocessing

This phase involved organizing data for training and testing the module. Datasets, initially in the form of flat files, were transformed into Python 3.12 programming by eliminating the zero-row values and filtering out noise by comparing them with the original sensor readings. Data distribution was balanced to prevent class imbalance issues.

We employed a 5-s sliding window with 50% overlap, segmenting data for deep learning model input. The collected data was partitioned into training, validation, and evaluation set in a 70:20:10 proportion, facilitating model training, hyperparameter tuning, and final assessment.

### 3.3 Feature extraction using gated recurrent neural networks

This section elucidates the GRN's role in feature extraction, beginning with an overview of RNN.

#### 3.3.1 Recurrent neural networks—An overview

In RNN, the hidden layer of every node is connected to the hidden layers of subsequent nodes in the interconnection. This architecture allows the nodes within the same hidden layer to be interconnected, facilitating the network's ability to perform time series and extensive data analysis by leveraging its capacity to remember and encode historical data swiftly. RNNs are adept at forming direct graph structures from node sequences, enabling the analysis of dynamic behaviors and sequence synchronization.

The internal memory (state) of the RNN plays a crucial role in processing input sequences, using past information to predict the future outcomes. However, in practical applications where there is a vital gap between the past and the future data, RNNs face challenges in retaining relevant historical information, leading to the well-known vanishing gradient problem. This issue undermines the network's ability to learn from long sequences, affecting the reliability of the results in real-time systems.

To address this limitation and enhance RNNs' performance, LSTM networks were brought into existence.

#### 3.3.2 Long short-term memory—An overview

**Figure 5:**
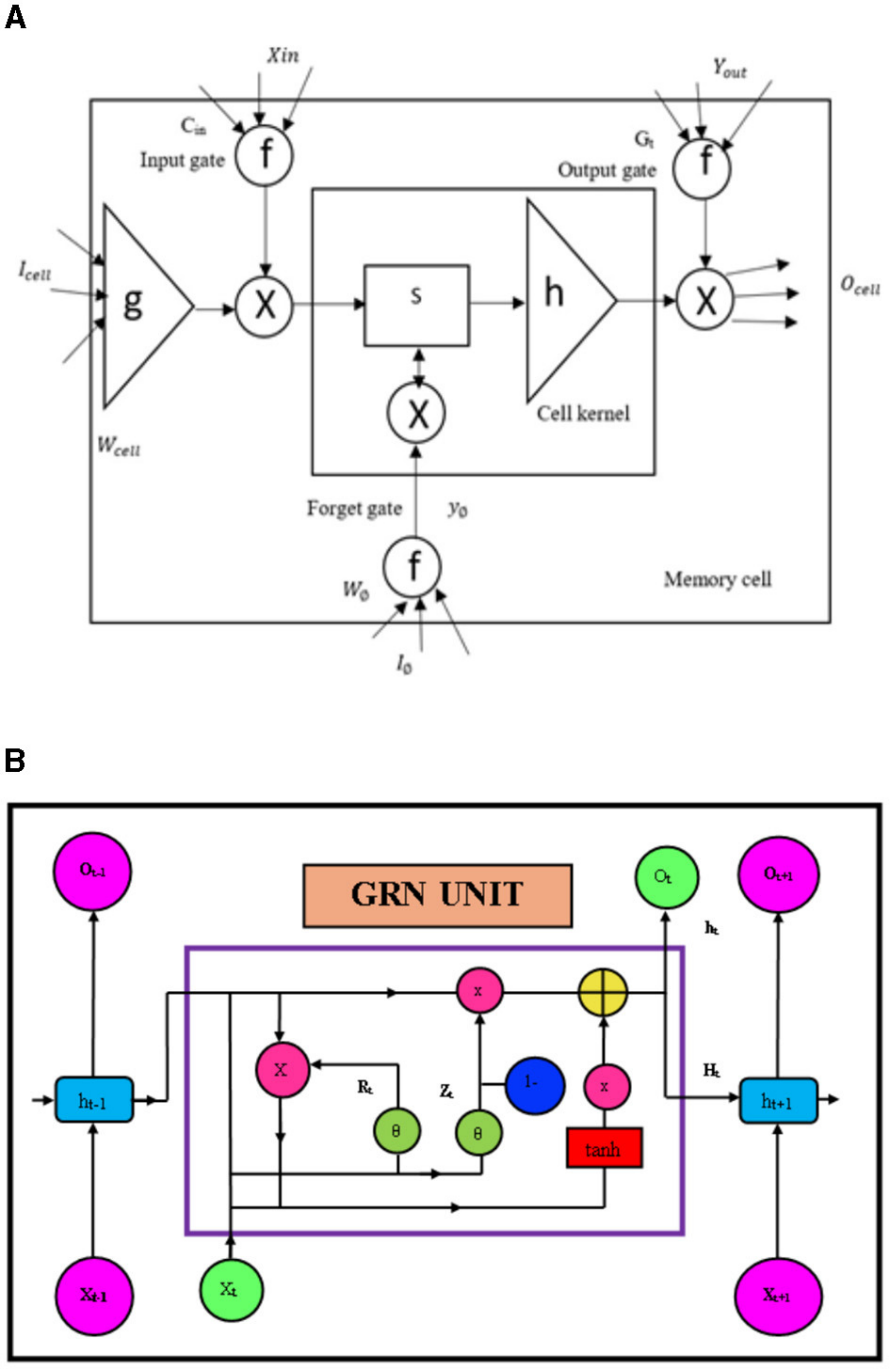
**(A)** LSTM structure; **(B)** GRN unit.

The LTSM networks are used for sequential processing problems, which are able to capture optimal temporal dependencies for a longer term. A typical LSTM network is presented in Figure 5.

The model discussed in this article proposes an innovative technique in addressing the given problem and considers a hybrid model that consists of an LSTM and an optimizer known as “whale.” An LSTM is depicted in Figure 5, which consists mainly of input gates, output gates, cell inputs, and forget gates. Let us assume that *x*_*t*_ is the input and “*h*_*t*_” is the output layer; thus, the previous corresponding output is “*h*__*t*_−1_.” Let us also assume that the state of the input cell is “*C*_*t*_” and the corresponding output state of the cell is “*G*_*t*_.” We also consider a previous state of “*G*_*t*_” is “*G*__*t*_−1_,” *j*_*t*_, *T*_*f*_, and *T*_0_ are assumed as the gates state. *G*_*t*_ and *h*_*t*_ are computed by using the following equations:


(1) $$\begin{array}{l} {I.G:\;j}_{t}=\;\theta \left(G_{l}^{i}.\;O_{t}+\;G_{h}^{i}.e_{t-1}+s_{i}\right)\; \end{array}$$

(2) $$\begin{array}{l} {\;F.G:\;T}_{f}\;=\;\theta \left(G_{l}^{f}.O_{t}+G_{h}^{f}.e_{t-1}+s_{f}\right)\; \end{array}$$

(3) $$\begin{array}{l} {O.G:\;T}_{0}=\;\theta \;\left(G_{l}^{0}.O_{t}+G_{h}^{0}.e_{t-1}+s_{0}\right) \end{array}$$

(4) $$\begin{array}{l} C.I:\;\tilde{T_{C}}=tanh\left(G_{l}^{C}.O_{t}+G_{h}^{C}.e_{t-1}+s_{C}\right) \end{array}$$

where $G_{l}^{0},\;G_{l}^{f}$, and $G_{l}^{i},G_{l}^{C}$ depicts the weight matrices among input gates and output layers and $G_{h}^{i},\;G_{h}^{f},\;G_{h}^{0}$, and $G_{h}^{C}$ indicates the weight conditions originated between hidden and input layers. The “*s*_*i*_, *s*_*f*_, *s*_0_, and *s*_*C*_ are the bias vectors, and tanh is considered to be a hyperbolic function”. The cell output state is computed as


(5) $$\begin{array}{l} {\;T}_{C}=k_{t}\times \;\tilde{T_{C}}+\;T_{f}\times T_{t-1} \end{array}$$

(6) $$\begin{array}{l} {\;e}_{t}=T_{0}\times \tanh\left(T_{C}\right) \end{array}$$

The ultimate result score is achieved through the aforementioned equation.

#### 3.3.3 Gated recurrent neural network

Figure 5A presents the GRN, which is responsible for long-term temporal feature extraction. The GRN has two main network components, LSTM and RNN (Priyanga et al., 2021). The GRN unit gets input data from the cloud system (Priyanga et al., 2021).

GRN is presented by the equation set below.


(7) $$\begin{array}{l} h_{t}=\left(1-x_{t}\right)\;⊙\;h_{t-1}+x_{t}\;⊙{\;h}_{t} \end{array}$$

(8) $$\begin{array}{l} \tilde{h_{t}}=g\left(W_{h}x_{t}+\;U_{h}\left(r_{t}\;⊙\;h_{t-1}\right)+\;b_{h}\right)\; \end{array}$$

(9) $$\begin{array}{l} z_{t}=\sigma \left(W_{h}x_{t}+U_{z}h_{t-1}+\;b_{z}\right)\; \end{array}$$

(10) $$\begin{array}{l} r_{t}=\sigma \left(W_{h}x_{t}+U_{r}h_{t-1}+\;b_{r}\right)\; \end{array}$$

The overall GRN characteristic equation is represented by


(11) $$P=GRU(\sum_{t=1}^{n}[x_{t,}h_{t,}z_{t,}\text{ and }r_{t}\left(W\left(t\right),B\left(t\right),\eta \left(tannh\right)\right)]\;$$

In the above equation, *x*_*t*_ represents the current state's input-feature and *y*_*t*_ is the corresponding output. *h*_*t*_ represents the current instants' module output. The update gate is depicted by *Z*_*t*_, whereas the reset gate is represented by *r*_*t*_; *W*(*t*) and *B*(*t*) are weight and bias weight, respectively, for the current instant.

### 3.4 Proposed GRN based class

Figure 5 illustrates the architecture of the proposed GRN networks integrated with the classification mechanism. The proposed network comprises an input layer, three GRN layers, and an output layer. The input layer consists of input sensor values from the IoT test beds. The three GRN layers are utilized to retrieve the time data for the sequence of sensor data. Each GRN layers are stacked in order to improve its stability and accuracy.

Each GRN layer has 44 hidden units and utilizes Leaky ReLU (L-ReLU) to enhance the robustness of the GRN algorithms. The output layers are constructed with the dense feedforward layers based on ELM. The intricate operational principles of the Extreme ELM are expounded upon. The depiction of input attribute maps within the ELM is symbolized by


(12) $$\begin{array}{l} S=F\left(G\left(i\right),P\right) \end{array}$$

where *F* is the GRN attributes gathered with the dimension P.

The output ELM function is denoted by


(13) $$\begin{array}{l} Y\left(i\right)=\;S\left(i\right)\beta =S\left(i\right)\;S^{T}{\left(\frac{1}{C}SS^{T}\right)}^{-1}O \end{array}$$

The overall training of ELM is given by


(14) $$T=\alpha (\sum_{i=1}^{N}\left(Y\left(i\right),\;B\left(i\right),W\left(i\right)\right)$$

where *Y*(*i*) is input feature maps; here the temporal matrices are represented by the symbol β . Usually, a Moore–Penrose generalized inverse theorem can be used to solve the temporal matrix problem. *Z*^*T*^ *is the inverse*. The symbols B and W represent the weights and bias factors, respectively. Finally, a SoftMax function is used to calculate the occurrence probability for each category, which is defined by Equation 15.


(15) $$Y^{\prime }=Softmax(T)$$

The forecasted outcome “*Y*” is employed to predict the DFU mechanism across established datasets, employing the cross-entropy function for the computation of the loss function is articulated through a mathematical expression, which can be paraphrased as follows:


(16) $$Loss=(\frac{1}{K})\sum_{i=1}^{K}(Y\left(i\right)\times log\;Y^{'}+\eta {\left|\left|\theta \right|\right|}^{2}$$

where *K* is the dimensional capsule feature length, η is the regularization co-efficient and |θ| is the constant.

### 3.5 Hyperparameter optimization

Hyperparameter optimization is the process of determining the best combination to tune the hyperparameters for obtaining the best performance in an adequate amount of time. This technique will also overcome the problem of overfitting, which maintains the stability of the model while training the large datasets. This research article employs the artificial raindrop algorithm (ARA) for model tuning to obtain the maximum performance from the network.

**Figure 6:**
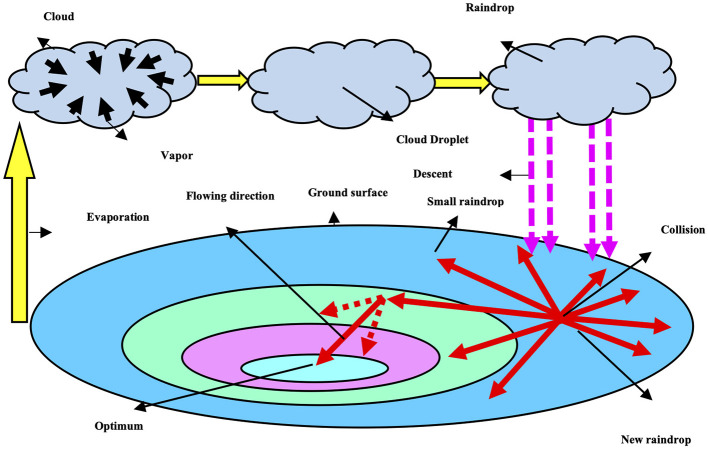
Artificial water drop optimization algorithm—its procedure.

We have used a heuristic algorithm, “artificial raindrop algorithm (ARA)” which is derived from the concept of natural rainfall system. Therefore, its steps resemble the natural rain process with several steps, including generation, falling, collision, like flowing of raindrops, which is a heuristic algorithm based on population. The major advantage of using ARA in the proposed network is to obtain less computational overhead, high-speed advantage, and less convergence time. Figure 6 represents the ARA algorithm. In the algorithm, as shown in the figure, the population consists of vapor, and a raindrop acts as an operator on the population. In Figure 6, vapor is denoted by a gray circle, which is a feasible solution. Raindrop is denoted by the blue circle, which is an operator. The population has five raindrop operators. The fitness function is represented by the altitude, which measures the effectiveness of a feasible solution. Table 4 presents the operators used in this optimization algorithm. Algorithm 1 presents the pseudo code of the ARA optimization algorithm.

**Table 4:** Distinct description of the primary raindrop operators.

**Name**	**Detailed definition**
Rain generation operation	$\phi GR\;\left(Pop\;\left(t\right)\right)=\left(\sum Ni\;=\;1\;\frac{Vapor_{i1}\left(t\right)}{N},\;\sum Ni=1\frac{Vapor_{i2}\left(t\right)}{N},\;\ldots ,\;\sum Ni\;=\;1\;\frac{{Vapor}_{i}\left(t\right)}{N}\right)$
Rain operation	$\phi DR\;(Raindrop\;\left(t\right))=Raindrop_{r2}\;\left(t\right)+\Phi^{*}(Raindrop_{r3}\;\left(t\right)-Raindrop_{r4}\;\left(t\right)),k\varepsilon \left\{1,2,\ldots ,N\right\};\Phi \epsilon (-1,1)$
Rain collision operation	ϕ*CR* (*New*_*Raindrop*_(*t*)∪*Pop* (*t*))
Rain flowing operator	ϕ*FR* (*Small*_*Raindrop*_(*t*)) = *Smal*_*l*_*Raindrop*_*i*_(*t*)+*d*(*t*, λ)
Vapor replacement operator	ϕ*RV* {*Pop* (*t*∪*Small*_*Raindrop*_(*t*))}

**Algorithm 1:**
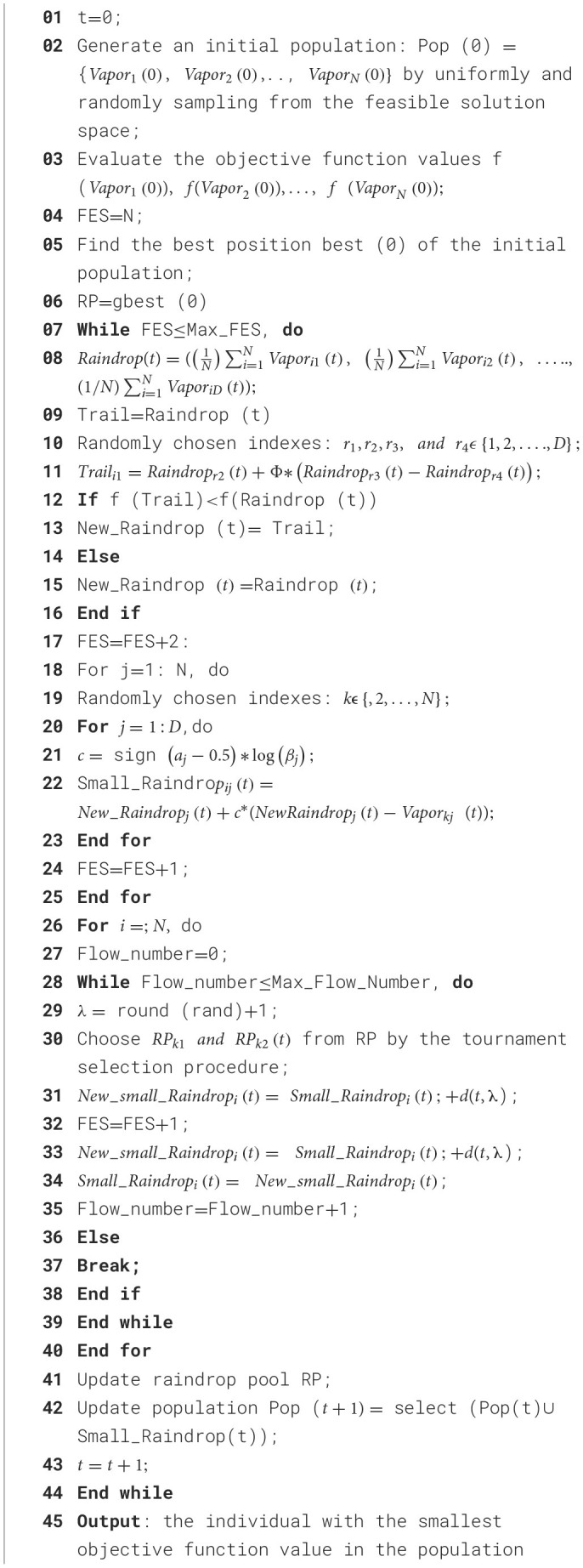
Input: N, the population size; D, the dimensions of optimization problem;τ , the flowing step parameter; RP, the raindrop pool; Max_Flow_Number, maximum number of flowing: Max_FES, maximum number of function evaluations.

The epochs, batch size, bias weights, momentum, hidden layers, and learning rate are the hyperparameters employed during the network's training process. Initially, these hyperparameters are chosen arbitrarily according to the AWDO and sent to the GRN dense training layer.

The fitness function of the suggested AWDO is depicted by the Equation 17. For every cycle, hyper-parameters are calculated by using Algorithm 1. The looping ends when the fitness function equates the Equation 17.


(17) $$\begin{array}{r} \text{Fitness Function}=\left(1-\text{Accuracy}\right)+\left(1-\text{Precision}\right)\\ +\left(1-\text{Recall}\right)+\left(1-\text{F1}-\text{score}\right)/4 \end{array}$$

For every cycle, the numerical hyperparameters are measured by utilizing the mathematical formulations mentioned in Algorithm 1. These parameters are then feed to network in which fitness function are calculated. If the fitness function reaches the limit, the looping will halt, or else it will persist indefinitely. In this method, AWDO exhibits a comparatively slower convergence rate in optimization tasks when contrasted with alternative meta-heuristic algorithms within the optimization timeframe, which will decrease and enhance the identification time. Algorithm 2 presents the complete pseudocode for the proposed hyperparameter optimization algorithm.

**Algorithm 2:**
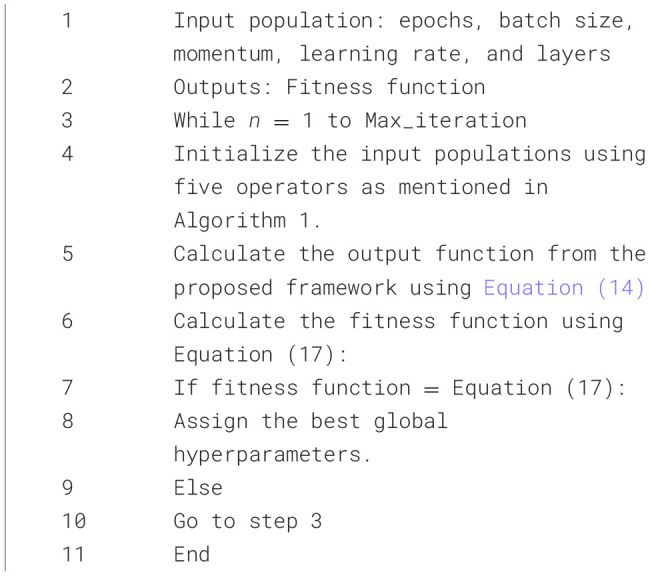
Pseudo-code for the hyperparameter optimization using AWDO.

## 4 Implementation details

**Figure 7:**
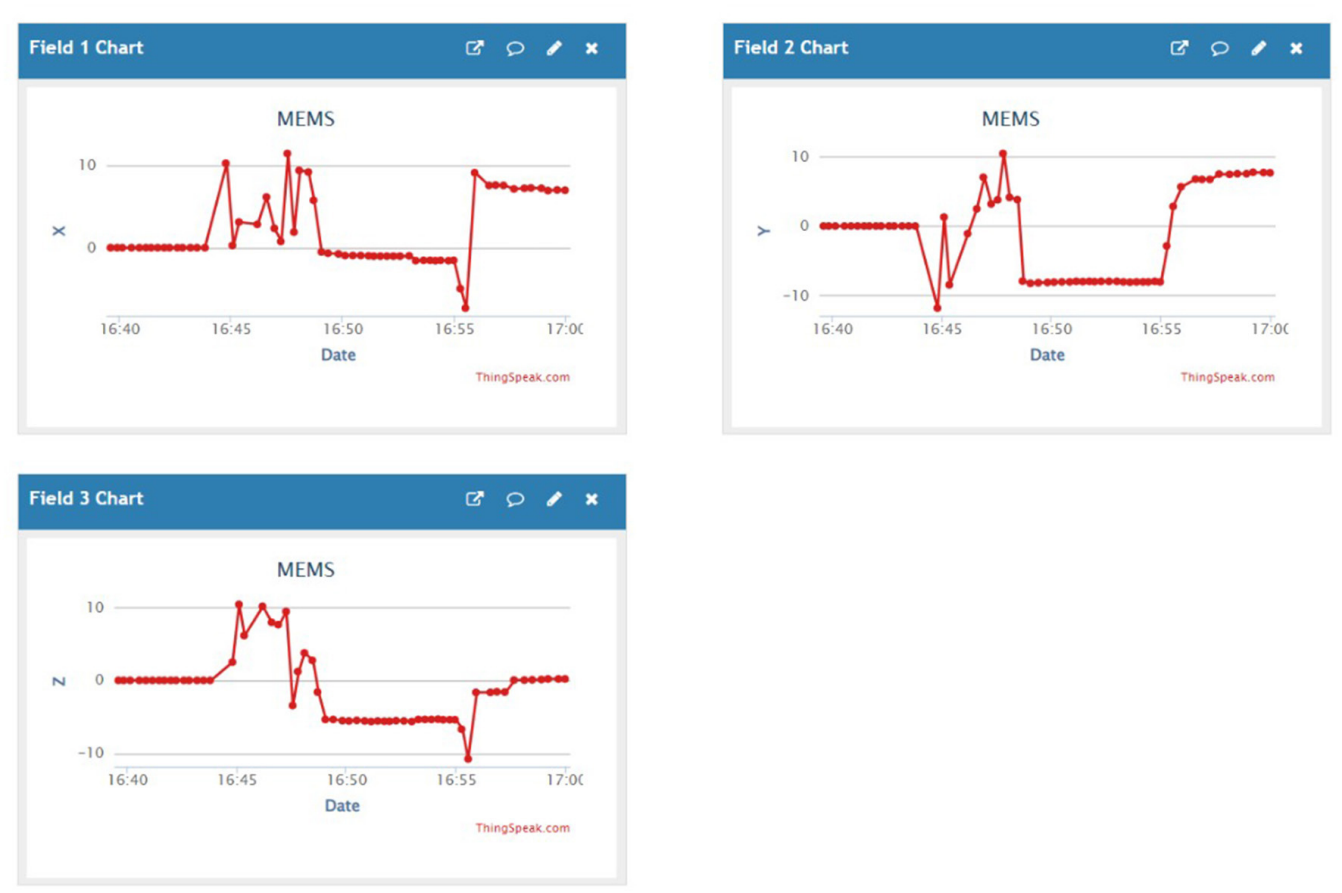
Accerolometers' data collected in the ThingSpeak Cloud, which are used for further analytics.

Experimental tests are conducted using TensorFlow with 14.76 GB RAM and NVIDIA Tesla T4 to generate and evaluate the results. IoT and HAR real-time datasets are used to assess the suggested framework. The description of data collection is depicted in Table 5. Figure 7 depicts the data collected from the real-time IoT test beds, which are stored in the ThingSpeak Cloud.

**Table 5:** Dataset used in validating the proposed method.

**Name of dataset**	**Number of samples**	**Cross-validation ratios (training: testing:validation)**
Real-time datasets	9.45.903	70:20:10
UCI HAR	8.40.258	80:10:10
mHealth	7.85.245	60:20:20

### 4.1 Model evaluation

Table 6 delineates the experimental parameters employed in training the suggested network. We have used different metrics for evaluation, including the F1-score, the area under the receiver operating characteristics (ROC), accuracy, recall, and confusion matrix. Table 7 presents the details of these performance metrics. Early stopping was used to conquer the overfitting and generalization issues in the training.

**Table 6:** Mathematical formulation for the performance metrics' calculation.

**S. No**.	**Performance metrics**	**Mathematical expression**
01	Accuracy	$\frac{\begin{array}{c} P+N \end{array}}{T+F+P+N}$
02	Recall	$\frac{\begin{array}{c} P \end{array}}{T+F}\times 100$
03	Specificity	$\frac{N}{N+P}$
04	Precision	$\frac{\begin{array}{c} N \end{array}}{T+P}$
05	F1-score	$2.\frac{Precison\times Recall}{Precision+Recall}$

**Table 7:** Performance metrics of the IPODTL-HAR framework in identifying the different activities of individuals with disabilities.

**Label activity HAR dataset**	**Performance metrics**				
	**Accuracy**	**Precision**	**Recall**	**Specificity**	**F1-score**
Walking	0.90	0.882	0.875	0.863	0.880
Sitting	0.892	0.885	0.865	0.854	0.874
Sleeping	0.895	0.873	0.843	0.853	0.862
Falling	0.90	0.893	0.872	0.882	0.872
Average value	0.892	0.873	0.865	0.853	0.879

**Figure 8:**
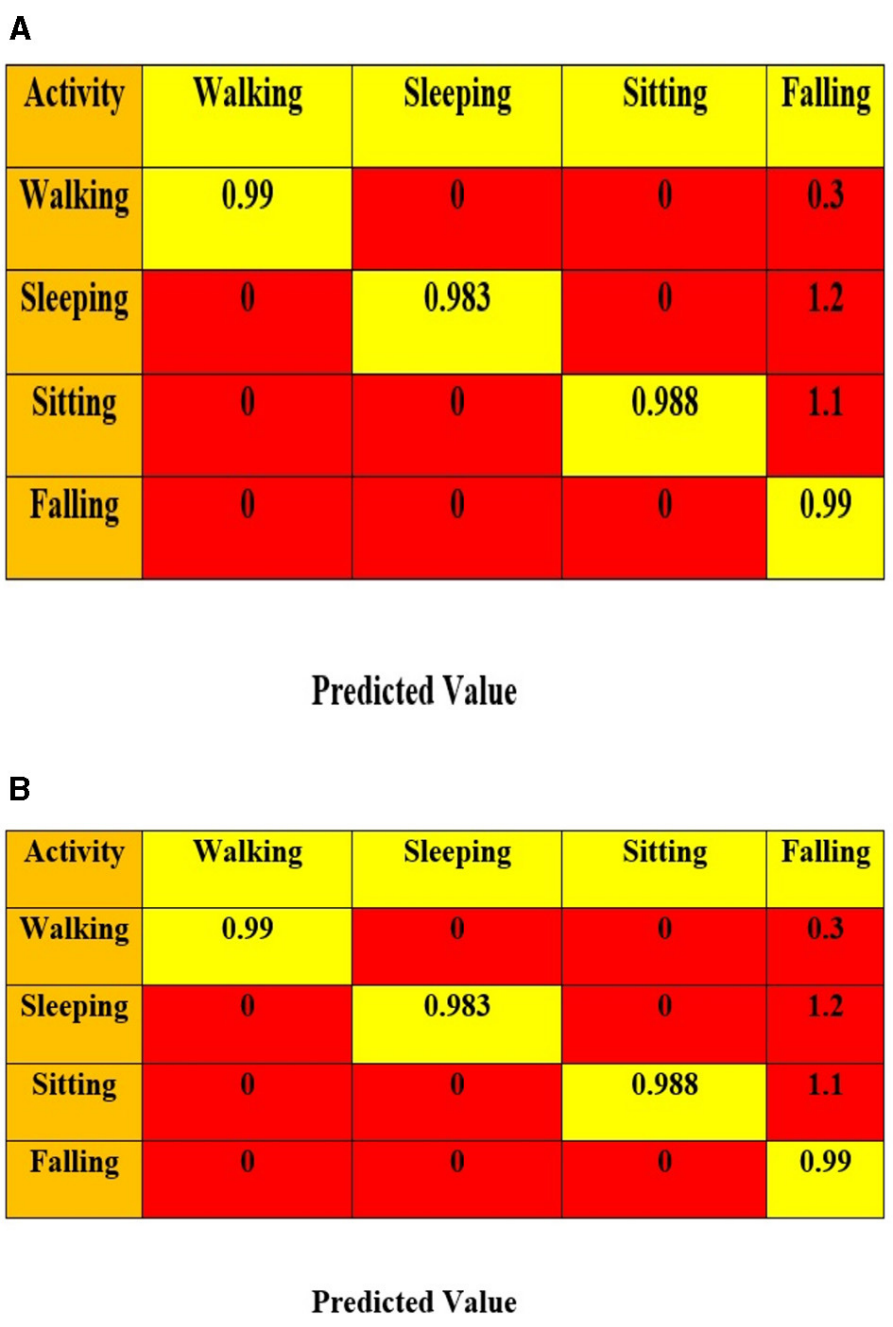
**(A, B)** Confusion matrix for the suggested framework using real-time training datasets and testing datasets.

This part calculates different experimentation evaluation metrics using real-time datasets. To exhibit the advancement of the suggested framework stands out significantly, the performance of the various other residing deep learning models is considered and compared with the model framework. The performance metrics of existing HAR systems, such as IPODTL-HAR, LSTM-HAR, GRN-HAR, 1D-CNN-HAR, and ANN, are calculated and compared with the framework. Figures 8A, B shows confusion matrix of the proposed algorithm in detecting the various activities of the elderly or individuals with disabilities.

Tables 7–12 present the efficacy of diverse learning methodologies in detecting the human activities. Table 7 illustrates the efficacy of the suggested model through its demonstration and seems to achieve utmost efficacy in categorizing human activities. The IPODTL–HAR model has produced the best performance in handling real-time data but is still less than the proposed model, which is evident from Table 8.

**Table 8:** Performance metrics of the proposed model in identifying the different activities of individuals with disabilities.

**Label activity HAR dataset**	**Performance metrics**				
	**Accuracy**	**Precision**	**Recall**	**Specificity**	**F1-score**
Walking	0.99	0.984	0.972	0.98	0.982
Sitting	0.983	0.975	0.970	0.971	0.972
Sleeping	0.992	0.983	0.979	0.982	0.988
Falling	0.990	0.982	0.979	0.983	0.981
Average value	0.99	0.982	0.976	0.975	0.982

**Figure 9:**
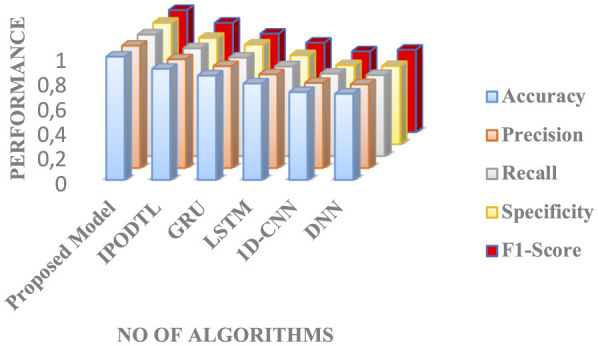
Comparative analysis of the performance of the various other models in identifying the HAR for individuals with disabilities.

Both GRU and LSTM models have produced moderate performance, whereas other existing algorithms, such as 1D-CNN and DNN, have produced the least performance, which is demonstrated in Tables 9–12. Figure 9 shows the comparative analysis between the average performances of different models. From Figure 9, it is evident that the incorporation of AWDO in feedforward classifier network ensemble with GRN networks has yielded the maximum performance. It is also proving its superiority in handling real-time datasets, which also proves to be a better choice for designing the intelligent HAR system that aids individuals with disabilities.

**Table 9:** Performance metrics of the GRU framework in identifying the different activities of individuals with disabilities.

**Label activity HAR dataset**	**Performance metrics**				
	**Accuracy**	**Precision**	**Recall**	**Specificity**	**F1-score**
Walking	0.843	0.821	0.79	0.83	0.802
Sitting	0.836	0.810	0.783	0.79	0.793
Sleeping	0.822	0.810	0.79	0.78	0.802
Falling	0.842	0.832	0.792	0.76	0.812
Average value	0.8375	0.818	0.79	0.80	0.792

**Table 10:** Performance metrics of the LSTM framework in identifying the different activities of individuals with disabilities.

**Label activity HAR dataset**	**Performance metrics**				
	**Accuracy**	**Precision**	**Recall**	**Specificity**	**F1-score**
Walking	0.712	0.70	0.673	0.653	0.683
Sitting	0.702	0.693	0.658	0.647	0.673
Sleeping	0.70	0.692	0.634	0.652	0.662
Falling	0.71	0.682	0.674	0.634	0.682
Average value	0.705	0.683	0.662	0.634	0.650

**Table 11:** Performance metrics of the ID–DNN model in identifying the different activities of individuals with disabilities.

**Label activity**	**Performance metrics**				
	**Accuracy**	**Precision**	**Recall**	**Specificity**	**F1-score**
Walking	0.684	0.674	0.653	0.632	0.662
Sitting	0.70	0.682	0.642	0.622	0.660
Sleeping	0.693	0.670	0.651	0.630	0.671
Falling	0.683	0.67	0.643	0.629	0.650
Average value	0.692	0.672	0.650	0.628	0.663

**Table 12:** Performance metrics of the DNN Model in identifying the different activities of individuals with disabilities.

**Label activity**	**Performance metrics**				
	**Accuracy**	**Precision**	**Recall**	**Specificity**	**F1-score**
Walking	0.783	0.77	0.727	0.763	0.75
Sitting	0.773	0.752	0.703	0.723	0.725
Sleeping	0.782	0.743	0.720	0.710	0.732
Falling	0.779	0.772	0.729	0.702	0.732
Average value	0.774	0.752	0.720	0.714	0.720

## 5 Conclusion and the future enhancement

In this research, we bring forth a pioneering hybrid deep learning-based HAR system specifically designed to recognize human activities for individuals with disabilities, thereby aiming to enhance their quality of life. Our proposed model innovates in feature extraction and activity classification within HAR frameworks. Utilizing GRN, our model establishes a novel feature extraction algorithm tailored for intricate activity patterns. Concurrently, we introduce a novel activity classifier, the DEFNN, refined through the AWDO algorithm.

The strategic amalgamation of DEFNN with AWDO optimizes the model's hyperparameters, enabling the deep feedforward networks to excel in classification accuracy by incorporating principles of ELMs. This methodological innovation ensures our HAR system not only achieves high precision in activity recognition but also maintains computational efficiency.

Our empirical analysis, grounded on real-time data gathered from IoT testbeds encompassing around 900,000 data points, underscores the model's robustness. Performance evaluations reveal our system outshines contemporary deep learning-based HAR frameworks, offering heightened detection performance and reduced computational load. Utilizing metrics for instance accuracy is 99.5%, precision is 98%, recall is 97%, specificity achieves 98%, and F1-score is 98.2%.

Looking forward, we aim to augment the system's capability by integrating edge analytics, mainly focusing on video and image inputs, to refine the HAR system further. This anticipated enhancement is poised to elevate the utility of our HAR system in assisting individuals with disabilities, potentially transforming their interaction with their environment and ensuring a higher quality of life.

AWDO with GRN to improve the performance of person recognition by reconstruction can be achieved using other block normalization methods and preprocessing steps of the dataset for reconstruction. Add conditions to create additional images that handle other changes in the image. The primary purpose of the performance is to achieve practical goals in the future work in the real-time settings.

## Data Availability

Publicly available datasets were analyzed in this study. This data can be found here: https://www.cis.fordham.edu/wisdm/dataset.php.

## References

[B1] Achirei S.-D. Heghea M.-C. Lupu R.-G. Manta V.-I. (2022). Human activity recognition for assisted living based on scene understanding. Appl. Sci. 12:10743. 10.3390/app12211074328661440

[B2] Alharbi A. Equbal K. Ahmad S. Ur Rahman H. Alyami H. (2021). Human gait analysis and prediction using the levenberg-marquardt method. Hindawi J. Healthc. Eng. 2021:11. 10.1155/2021/5541255 33680414 PMC7906803

[B3] Alotaibi F. Alnefaie M. Al-Wesabi F. Alduhayyem M. Hilal A. Hamza M. (2023). Optimal deep recurrent neural networks for iot-enabled human activity recognition in elderly and disabled persons. J. Disab. Res. 2:23. 10.57197/JDR-2023-0023

[B4] Arzani M. M. Fathy M. Azirani A. A. Adeli E. (2021). Switching structured prediction for simple and complex human activity recognition. IEEE Trans. Cybernet. 51, 5859–5870. 10.1109/TCYB.2019.2960481 31945007

[B5] Chen K. Zhang D. Yao L. Guo B. Yu Z. Liu Y. (2020). Deep learning for sensor-based human activity recognition: overview, challenges and opportunities. arXiv [preprint] arXiv:2001.07416. 10.48550/arXiv.2001.0741639124092

[B6] Choudhury N. A. Soni B. (2023). Enhanced complex human activity recognition system: a proficient deep learning framework exploiting physiological sensors and feature learning. IEEE Sens. Lett. 7, 1–4. 10.1109/LSENS.2023.332612637529707

[B7] Dahal A. Moulik S. (2024). The multimodel stacking and ensemble framework for human activity recognition. IEEE Sensors Lett. 8, 1–4. 10.1109/LSENS.2024.3451960

[B8] Duhayyim M. A. (2023). Parameter-tuned deep learning-enabled activity recognition for disabled people. Comp. Mater. Continua 75, 6587–6603. 10.32604/cmc.2023.033045

[B9] Fotouhi H. G. Chour H. Benmabrouk M. (2022). “Human daily activities from detection to prediction,” in 2022 45th Jubilee International Convention on Information, Communication and Electronic Technology (MIPRO) (Opatija: MIPRO), 985–990.

[B10] Ha S. Choi S. (2016). “Convolutional neural networks for human activity recognition using multiple accelerometer and gyroscope sensors,” in 2016 *International Joint Conference on Neural Networks* (Vancouver, BC: IEEE), 381–388.

[B11] Helmi A. M. Al-Qaness M. A. Dahou A. Damaševičius R. Krilavičius T. Elaziz M. A. (2021). A novel hybrid gradient-based optimizer and grey wolf optimizer feature selection method for human activity recognition using smartphone sensors. Entropy 23:1065. 10.3390/e23081065 34441205 PMC8393762

[B12] Huang G.-B. Zhu Q.-Y. Siew C.-K. (2006). Extreme learning machine: theory and applications. Neurocomputing 70:489–501, 10.1016/j.neucom.2005.12.126

[B13] Jaén-Vargas M. Leiva K. M. R. Fernandes F. Gonçalves S. B. Silva M. T. Lopes D. S.. (2022). Effects of sliding window variation in the performance of acceleration-based human activity recognition using deep learning models. PeerJ. Comp. Sci. 8:e1052. 10.7717/peerj-cs.1052 36091986 PMC9455026

[B14] Jiang W. Yin Z. (2015). “Human activity recognition using wearable sensors by deep convolutional neural networks,” in Proceedings of the 23rd ACM International Conference on Multimedia (New York: ACM), 1307–1310.

[B15] Jiao W. Zhang C. (2023). An efficient human activity recognition system using WiFi channel state information. IEEE Syst. J. 17, 6687–6690. 10.1109/JSYST.2023.3293482

[B16] Karayaneva Y. Sharifzadeh S. Jing Y. Tan B. (2023). Human activity recognition for ai-enabled healthcare using low-resolution infrared sensor data. Sensors 23:478. 10.3390/s23010478 36617075 PMC9824082

[B17] Kushwaha A. K. Kar A. K. Dwivedi Y. K. (2021). Applications of big data in emerging management disciplines: a literature review using text mining. Int. J. Inform. Manage. Data Insights 1:100017. 10.1016/j.jjimei.2021.100017

[B18] Laput G. Harrison C. (2019). “Sensing fine-grained hand activity with smartwatches,” in Proceedings of the 2019 CHI Conference on Human Factors in Computing Systems (New York: ACM), 338.

[B19] Lester J. Choudhury T. Kern N. Borriello G. Hannaford B. (2005). “A hybrid discriminative/generative approach for modeling human activities,” in IJCAI'05: Proceedings of the 19th International Joint Conference on Artificial Intelligence.27534393

[B20] Mekruksavanich S. Jitpattanakul A. (2021). Deep convolutional neural network with RNNs for complex activity recognition using wrist-worn wearable sensor data. Electronics 10:1685. 10.3390/electronics10141685

[B21] Mihoub A. (2021). A deep learning-based framework for human activity recognition in smart homes, mobile information *Systems*. 2021:11. 10.1155/2021/6961343

[B22] Moon J. Anh Le N. Minaya N. H. Choi S. (2020). Multimodal Few-Shot Learning for Gait Recognition. Appl. Sci. 10, 7619; 10.3390/app10217619

[B23] Munoz-Organero M. Ruiz-Blazquez R. (2017). Time-elastic generative model for acceleration time series in human activity recognition. Sensors 17:2. 10.3390/s17020319 28208736 PMC5336010

[B24] Nasir J. A. Khan O. S. Varlamis I. (2021). Fake news detection: a hybrid CNN-RNN based deep learning approach. Int. J. Inform. Manage. Data Insights 1:100007. 10.1016/j.jjimei.2020.100007

[B25] Nweke H. F. Teh Y. W. Al-garadi M. A. Alo U. R. (2018). Deep learning algorithms for human activity recognition using mobile and wearable sensor networks: state of the art and research challenges. Expert Syst. Appl. 105, 233–261. 10.1016/j.eswa.2018.03.056

[B26] Pramanik R. Sikdar R. Sarkar R. (2023). Transformer-based deep reverse attention network for multi-sensory human activity recognition. Eng. Appl. Artif. Intellig. 122:106150, 10.1016/j.engappai.2023.106150

[B27] Priyanga P. Pattankar V. V. Sridevi S. (2021). A hybrid recurrent neural network-logistic chaos-based whale optimization framework for heart disease prediction with electronic health records. Comput. Intellig. 37:1. 10.1111/coin.12405

[B28] Ramasamy Ramamurthy S. Roy N. (2018). Recent trends in machine learning for human activity recognition a survey. Wiley Interdiscipl. Rev. 8, 1–11. 10.1002/widm.1254

[B29] Ranasinghe D. C. Torres R. L. S. Wickramasinghe A. (2013). “Automated activity recognition and monitoring of elderly using wireless sensors: Research challenges,” in Proceedings of the 2013 5th IEEE International Workshop on Advances in Sensors and Interfaces (Bari: IEEE), 224–227.

[B30] Ranasinghe S. Al MacHot F. Mayr H. C. (2016). A review on applications of activity recognition systems with regard to performance and evaluation. Int. J. Distrib. Sensor Netw. 12:8. 10.1177/1550147716665520

[B31] Roy B. Cheung H. (2018). “A deep learning approach for intrusion detection in internet of things using bi-directional long short-term memory recurrent neural networks,” in IEEE International Telecommunication Conference (Sydney, NSW: IEEE).

[B32] Saleh A. M. Hamoud T. (2021). Analysis and best parameters selection for person recognition based on gait model using CNN algorithm and image augmentation. J. Big Data 8:1 10.1186/s40537-020-00387-6 33425651 PMC7778727

[B33] Sangeetha G. Shantha Kumar S. Harshavardhan S. Varun D. (2023). Human activity recognition using dnn classifier and feature analysis. Int. J. Creat. Res. Thoug. 11, f24–f30. Available at: https://www.ijcrt.org/papers/IJCRT2303572.pdf

[B34] Sharma G. Dhall A. Subramanian R. (2024). MARS: a multiview contrastive approach to human activity recognition from accelerometer sensor. IEEE Sensors Lett. 8, 1–4. 10.1109/LSENS.2024.3357941

[B35] Shen Y. H. He K. X. Zhang W. Q. (2018). “SAM-GCNN: a gated convolutional neural network with segment-level attention mechanism for home activity monitoring,” in 2018 IEEE International Symposium on Signal Processing and Information Technology (ISSPIT) (Louisville, KY: IEEE), 679–684. 10.1109/ISSPIT.2018.8642767

[B36] Uddin M. Z. Soylu A. (2021). Human activity recognition using wearable sensors, discriminant analysis, and long short-term memory-based neural structured learning. Sci Rep. 11:16455. 10.1038/s41598-021-95947-y 34385552 PMC8361103

[B37] Wang B. Huang S. Qiu J.. (2015). Parallel online sequential extreme learning machine based on MapReduce. Neurocomputing 149, 224–232. 10.1016/j.neucom.2014.03.076

[B38] Wang S. Zhang L. Wang X. Huang W. Wu H. Song A. (2024). Patchhar: A mlp-like architecture for efficient activity recognition using wearables. IEEE Trans. Biomet. Behav. Identity Sci. 6, 169–181. 10.1109/TBIOM.2024.3354261

[B39] Xin B. Wang F. Zhai Z. (2021). Balwin-teaching-learning-based artificial raindrop algorithm for UAV route planning. Mathem. Probl. Eng. 2021:8865403, 14. 10.1155/2021/8865403

[B40] Yao L. Guo B. Yu Z. Liu Y. (2021). Deep learning for sensorbased human activity recognition: overview, challenges, and opportunities. ACM Comp. Surv. 54:4. 10.1145/344774439124092

[B41] Yin Y. Xie L. Jiang Z. Xiao F. Cao J. Lu S. (2024). A systematic review of human activity recognition based on mobile devices: overview, progress and trends. IEEE Commun. Surv. Tutor. 26, 890–929. 10.1109/COMST.2024.3357591

[B42] Zhang J. Liu Y. Yuan H. (2023). Attention-based residual BiLSTM networks for human activity recognition. IEEE Access. 99:1. 10.1109/ACCESS.2023.3310269

[B43] Zhang Z. Yang Y. Lv Z. Gan C. Zhu Q. (2021). LMFNet: human activity recognition using attentive 3-D residual network and multistage fusion strategy. IEEE Intern. Things J. 8, 6012–6023. 10.1109/JIOT.2020.3033449

[B44] Zhu C. Sheng W. (2012). Realtime recognition of complex human daily activities using human motion and location data. IEEE Trans. Biomed. Eng. 59, 2422–2430. 10.1109/TBME.2012.2190602 22434793

